# Effects of Pollen Germination and Pollen Tube Growth under Different Temperature Stresses in Mango (*Mangifera indica* L.) by Metabolome

**DOI:** 10.3390/metabo14100543

**Published:** 2024-10-11

**Authors:** Xinyu Liu, Lirong Zhou, Chengxun Du, Songbiao Wang, Hongjin Chen, Wentian Xu, Zhuanying Yang, Qingzhi Liang

**Affiliations:** 1 College of Coastal Agricultural Sciences, Guangdong Ocean University, Zhanjiang 524088, China; 2 Key Laboratory of Disaster Weather Defense and Climate Resource Utilization of Panzhihua City, Renhe Meteorological Bureau of Panzhihua, Panzhihua 617000, China; 3 South Subtropical Crops Research Institute, Chinese Academy of Tropical Agricultural Sciences, Zhanjiang 524091, China

**Keywords:** mango, metabolomics, pollen activity, temperature stress

## Abstract

Background: The dramatic temperature fluctuations spurred by global warming and the accompanying extreme weather events inhibit mango growth and threaten mango productivity. Particularly, mango flowering is highly sensitive to temperature changes. The mango fruit setting rate was significantly positively correlated with pollen activity, and pollen activity was regulated by different metabolites. Methods: In this study, the in vitro pollen of two mango varieties (‘Renong No.1’ and ‘Jinhuang’), in which sensitivity to temperature differed significantly, were subjected to different temperature stresses (15 °C, 25 °C and 35 °C), and their metabolomics were analyzed. Results: The present results showed that 775 differential metabolites were screened by liquid chromatography–mass spectrometry and divided into 12 categories. The two varieties had significant differences in metabolite expression under different temperature stresses and the effect of low temperature on ‘Renong No.1’ mainly focused on amino acid metabolism, while the effect on ‘Jinhuang’ was mainly related to glycolysis. However, under the 35 °C temperature stress, ‘Renong No.1’ responded by redistributing riboflavin and betaine in vivo and the most obvious metabolic pathway of ‘Jinhuang’ enrichment was pyrimidine metabolism, which had undergone complex main body formation and extensive regulatory processes. The changes of metabolites of different varieties under low temperature and high temperature stress were different. Among them, flavonoids or flavonoid derivatives were included in class A (216 metabolites), C (163 metabolites) and D (233 metabolites) metabolites, indicating that flavonoid metabolites had an obvious regulatory effect on mango pollen metabolism under different temperature stress. Conclusions: The present results provide valuable information for reproductive biology studies and breeding in mango, in particular, the selection and breeding of the most suitable varieties for different production areas.

## 1. Introduction

Mango (*Mangifera indica* L., 2n = 40) is an important tropical and subtropical fruit that belongs to the genus Mangifera in the family Anacardiaceae. [1]. Mango is highly regarded as one of the five famous fruits in the world. It is distributed in almost all tropical and subtropical regions across the world [2]. Mangoes were native to the tropical areas of Southeast Asia. At present, mangoes are grown in more than 100 countries and regions around the world, distributed between 30° north and south latitude. India has the largest area under mango cultivation, accounting for 40%, followed by China with about 17% [3,4]. According to historical records, mangoes cultivated in China were introduced from Southeast Asia and the history of mango cultivation in China can be traced back as far as 1370 years ago [5,6]. There were mainly five mango species in China, and about 200 cultivars of mango were widely cultivated [7,8].

As an important factor controlling plant growth and development, temperature has a serious impact on plant flowering [9]. Many studies have shown that temperature stress usually leads to pollen abortion and asynchrony in pollen and stigma development [10]. In particular, high temperatures affects pollen germination, pollen tube growth, fertilization, flower shedding and seed setting [11,12]; low temperature can also inhibit pollen germination and reduce the plant fruit setting rate [13,14]. In addition, extreme high temperature weather has become more and more frequent due to global climate change in recent years [15], which seriously affects the pollen germination and fruit setting yield at the flowering stage [16,17]. It is estimated that Earth’s surface temperature will rise by 1.4~5.8 °C by 2100 [18] and these effects will directly result in serious economic losses. Therefore, the influence of temperature on pollen germination has attracted more and more attention. To ensure the sustainability of agriculture, it is necessary to identify and distinguish the response and tolerance of mango pollen activity under different temperature stresses.

Pollen grain is the sexual reproductive unit and carrier of male genetic material in higher plants, which plays an important role in reproductive processes. The formation of the pollen tube is a good and simple standard to judge the growth and development of pollen [19]. Pollen development, specifically, represents a very narrow developmental window. It is a complex process that requires the coordinated activity of different gametophytic and sporophytic cell types and tissues, making it particularly sensitive to environmental challenges. Developing pollen is situated among the plant structures that are most sensitive to high temperatures, and a decrease in pollen viability is often associated with an alteration of metabolite content [20,21]. In the process of pollen development, metabolites can not only play a role in pollen nutrition or signal transduction, but also serve as protective agents against environmental stress. Previous studies showed that flavonoids and polyamines can act as scavengers of reactive oxygen species (ROS) [22,23]; metabolites such as lipids, flavonoids and polyamines were also involved in the development of the pollen wall, including different coating layers (such as the outer wall, inner wall and sporopollenin), playing an important role in protecting pollen from abiotic stress [24,25]. However, the current understanding of the role and importance of metabolites in mango pollen development under different temperature stresses is still limited.

Previous studies on metabolic changes during pollen development always focused on the detection of a limited set of target compounds. In recent years, the use of mass spectrometry–based metabonomics methods, such as gas chromatography–mass spectrometry (GC-MS) and liquid chromatography–mass spectrometry (LC-MS), has made it possible to simultaneously detect hundreds to thousands of metabolites in a single extract, which provides a more comprehensive understanding of all aspects of plant development and stress response [26,27], and there are more and more studies on mango pollen developmental biology and pollination [28]. Most of these studies focus on the morphological, ecological and cytological characteristics of genetic variation. However, what are the mechanisms underlying differences in tolerance to high and low temperature stresses during flowering in different mango genotypes? The metabolomics analysis of different mango pollen under different temperature stresses during in vitro germination has not been studied. This research will increase our understanding regarding the relationship between the pollen activities under different temperature stresses in mango by metabolome.

## 2. Materials and Methods

### 2.1. Pollen Collection

In this study, the in vitro pollen of two mango varieties (‘Renong No.1’ and ‘Jinhuang’), in which sensitivity to temperature differed significantly, were subjected to different temperature stresses (15 °C, 25 °C and 35 °C) for effects analysis of pollen germination and pollen tube growth (Table 1). Mango pollen was collected from the mango planting resource nursery of the South Subtropical Crop Research Institute, Chinese Academy of Tropical Agricultural Sciences, in March 2023 (Zhanjiang city, China). Mango trees of each variety were randomly selected at the full flowering stage (the number of flowers accounted for 15~75% of the total flowers). The fresh anthers opened on that day were put into the culture medium and quickly brought back to the laboratory for observation and further analysis under different temperature stresses after full shaking.

### 2.2. Pollen Germination and Pollen Tube Length in Vitro

(1) Mango pollen culture medium preparation: 150 mg boric acid, 6.0 mg calcium sulfate, 100 mg magnesium sulfate, 100 mg potassium nitrate and 250 g polyethylene glycol were mixed, and they were dissolved to 1 L, then the mixture was heated and boiled to fully dissolve, and they were stored at room temperature after cooling.
(2) Pollen collection and pretreatment: the prepared culture medium was packed into 500 ul centrifuge tubes, then 12–15 anthers were randomly taken from each centrifuge tube, and then were shook sufficiently to make the pollen on the anther adhere to the culture medium.
(3) Pollen culture in vitro at different temperatures: the centrifuge tubes were stored in incubators with a relative humidity of 60% (Lvbo, Model RTOP-500Y, Hangzhou, China) at 15 °C, 25 °C and 35 °C for 3 h temperature stress, respectively, and three replicates for each treatment.
(4) Determination of pollen germination rate under different temperature conditions: a clean concave slide was taken and 1–2 drops of evenly mixed culture solution was put into the concave for observation. Each treatment was repeated three times, five visual fields were randomly selected for each repetition, and a total of 200~500 pollen grains were observed. The pollen germination standard was that the pollen tube length exceeded the pollen grain diameter. The number of germinated pollen and the total number of pollen at different temperature gradients were observed, and the germination rate was counted.

The calculation formula is as follows:

(1) $$Q=\frac{w}{W}\times 100\%$$

where Q is the pollen germination rate; *w* is the number of germinated pollen; and W is the total pollen.

### 2.3. Metabolite Extracton

#### 2.3.1. Sample Preparation and Extraction

Biological samples (three replicates for each treatment) were freeze-dried by a vacuum freeze-dryer (Scientz-100F) (Ningbo Xinzhi Biotechnology Co., Ltd., Ningbo city, China). The freeze-dried sample was crushed using a mixer mill (MM 400, Retsch) with a zirconia bead for 1.5 min at 30 Hz. Then, 100 mg of lyophilized powder was dissolved with 1.2 mL 70% methanol solution, the mixture was vortexed 30 s every 30 min for 6 times and the sample was placed in a refrigerator at 4 °C overnight. Following centrifugation at 12,000 rpm for 10 min, the extracts were filtrated (SCAA-104, 0.22 μm pore size; ANPEL, Shanghai, China, http://www.anpel.com.cn (accessed on 13 September 2024)) before UPLC-MS/MS analysis.

#### 2.3.2. UPLC Conditions

The UPLC-ESI-MS/MS system was employed to analyze the sample extracts (UPLC, SHIMADZU NexeraX2, www.shimadzu.com.cn/ (accessed on 13 September 2024); MS, Applied Biosystems 4500 Q TRAP, www.appliedbiosystems.com (accessed on 13 September 2024)). The following analysis conditions were used for analysis: UPLC: column, Agilent SB-C18 (1.8 µm, 2.1 mm × 100 mm); Solvent A (pure water containing 0.1% formic acid) and solvent B (acetonitrile containing 0.1% formic acid) combined to form the mobile phase. Samples were measured using a gradient procedure with starting conditions of 95% A, 5% B. Over a period of 9 min, the program sets the linear gradient to 5% A, 95% B and holds the 5% A, 95% B component for 1 min. The composition was then adjusted to 95% A, 5.0% B over 1.10 min and held for 2.9 min. The flow rate was set at 0.35 mL per minute, the column temperature was set at 40 °C and the injection volume was 4 microlitres. The effluent was connected to the ESI-triple tetraphon linear ion trap (QTRAP)-MS.

#### 2.3.3. ESI-Q TRAP-MS/MS

LIT and Triple Quadrupole (QQQ) scans were obtained on a Triple Quadrupole Linear Ion Trap Mass Spectrometer (Q TRAP), an AB4500 Q TRAP UPLC/MS/MS system equipped with an ESI Turbo Ion Spray interface operating in both positive and negative ion modes, controlled by Analyst 1.6.3 software (AB Sciex, Victoria, Australia).

The ESI source runs with the following parameters: source temperature 550 °C; ion spray voltage (IS) 5500 V (positive ion mode)/−4500 V (negative ion mode); ion source gas I (GSI), gas II (GSII), curtain gas (CUR); ion source, turbo spray, which were set at three levels, respectively, namely 50, 60 and 25.0 psi; meanwhile, the collision-activated dissociation (CAD) was high.

Under QQQ and LIT modes, 10 and 100 μmol/L polyphol-propylene glycol solutions were used for instrument adjustment and quality calibration, respectively. When the collision gas (nitrogen) was set to medium, it obtains QQQ scanning in the form of MRM experiments. Further optimization of DP and CE was conducted for individual MRM transitions. We monitored a set of specific MRM transitions in each period according to the metabolites that were eluted during this period.

### 2.4. Metablolic Profiling

#### 2.4.1. PCA Analysis

Unsupervised PCA (principal component analysis) was executed by statistical functions in R (www.r-project.org) (accessed on 26 March 2024) (Software: R (base package), version: 3.5.1). Before conducting an unsupervised PCA analysis, the unit variance was scaled for the data.

#### 2.4.2. Hierarchical Cluster Analysis and Pearson Correlation Coefficients

The results of the HCA (hierarchical cluster analysis) of the sample and metabolites are shown in the form of heatmaps with dendrograms, while the Pearson correlation coefficients (PCCs) between samples are calculated and presented in the form of heatmaps by the core function of R. Both HCA and PCC were implemented by the Heatmap of R package (Software: R (base package; Hmisc), version: 3.5.1; 4.4.0). In HCA, the normalized signal intensity of metabolites (unit variance scaling) is presented in the form of a color spectrum.

#### 2.4.3. Differential Metabolites Selected

The significant criteria for regulating metabolites between groups was set by VIP ≥ 1 and absolute log_2_FC (fold change) ≥ 1. The R software package Metaboanalystr (version: 1.0.1) was used to extract the VIP value from the partial least squares–discriminant analysis (OPLS-DA) result (Software: R (MetaboAnalystR), version: 1.0.1). The result also included score plots and permutation plots. Before OPLS-DA, the data were log transform (log_2_) and mean centering. In order to avoid excessive fitting, a permutation test (200 permutations) was carried out.

#### 2.4.4. KEGG Annotation and Enrichment Analysis

The identified metabolites were annotated using the KEGG Compound Database (https://www.kegg.jp/kegg/compound/ (accessed on 13 September 2024)) and then the annotated metabolites were mapped to the KEGG Pathway Database (https://www.kegg.jp/kegg/pathway.html (accessed on 13 September 2024)). Pathways mapped to significantly regulated metabolites were then fed into MSEA (metabolite sets enrichment analysis), where the significance was determined by the hypergeometric test’s *p*-values.

## 3. Results

### 3.1. Changes in the Pollen Germination Rate under Different Temperature Stress

In the process of plant growth and development, pollen germination is very sensitive, especially to temperature at the flowering period. Temperature stress will cause delay or an early flowering period or pollen abortion. Temperature stress often leads to pollen abortion and asynchronous development between pollen and the ovary, because pollen is more sensitive to temperature than the stigma.

**Figure 1:**
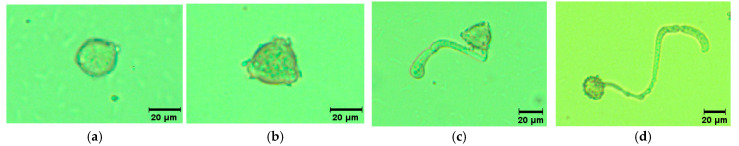
The germination state of pollen: (**a**) no germination, (**b**) ready to germinate, (**c**) germinating, (**d**) germinated.

**Figure 2:**
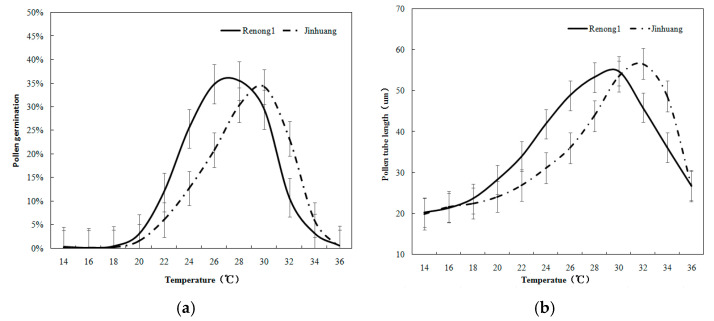
(**a**) The pollen germination rate and (**b**) pollen tube length growth of ‘Renong No. 1’ and ‘Jinhuang’ genotypes in response to different temperature stress.

The pollen germination rate and pollen tube length growth were analyzed (Figure 1) by observing the process of pollen germination. Preliminary experiment results on the pollen germination and pollen tube length under different temperature stresses indicated the following: the pollen germination and pollen tube length of ‘Renong No.1’ and ‘Jinhuang’ reached the maximum at 24 °C–26 °C; and the pollen did not germinate under 15 °C, and then rose with the increase in temperature, reaching the maximum at 25 °C, with the average germination rates of ‘Renong No.1’ and ‘Jinhuang’ pollen being 30.1% and 27.8%, respectively, and then decreased, even below 10% when above 35 °C (Figure 2a). The growth of the pollen tube also showed a similar trend, from the average value of 45.1 μm at 25 °C dropping to 34.7 μm at 35 °C (Figure 2b).

Based on the results of our previous research, both low temperature and high temperature had obvious inhibitory effects on mango pollen germination in vitro [29]. When the temperature was between 22 °C and 32 °C, the pollen tube grew well, and the average length reached 3–4 times of the pollen diameter. Higher than 32 °C or lower than 22 °C inhibited pollen germination, which may eventually affect mango pollination and fruit setting.

**Figure 3:**
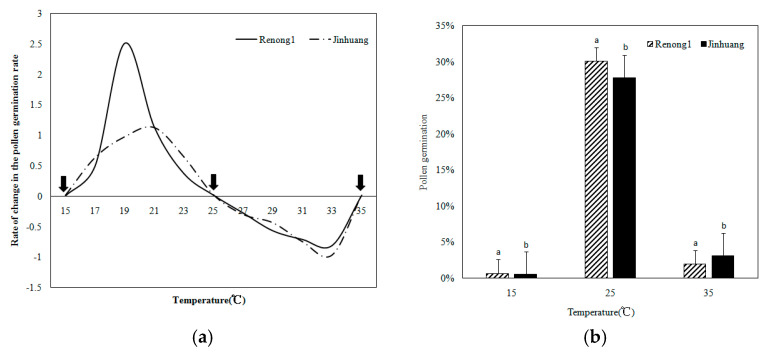
(**a**) The changes in the pollen germination rate of two varieties of mango at different temperature stresses and (**b**) the pollen germination of ‘Renong No.1’ and ‘Jinhuang’ in response to temperature. The lowercase letters on bars represents the levels of significance. Means with the same lowercase letters at the top of the bar do not differ significantly at *p* < 0.05.

As shown in Figure 3a, the present results indicate the changes in the pollen germination rate of two varieties of mango at different temperatures (15 °C, 25 °C and 35 °C). When the temperature was as low as 15 °C, the pollen did not germinate; however, when the temperature was as high as 35 °C, it inhibited pollen germination, and the pollen germination rate decreased to less than 3. These results are shown in Figure 3b and Figure S1.

### 3.2. Metabolites Variations in ‘Renong No.1’ and ‘Jinhuang’ under Different Temperature Stress

**Figure 4:**
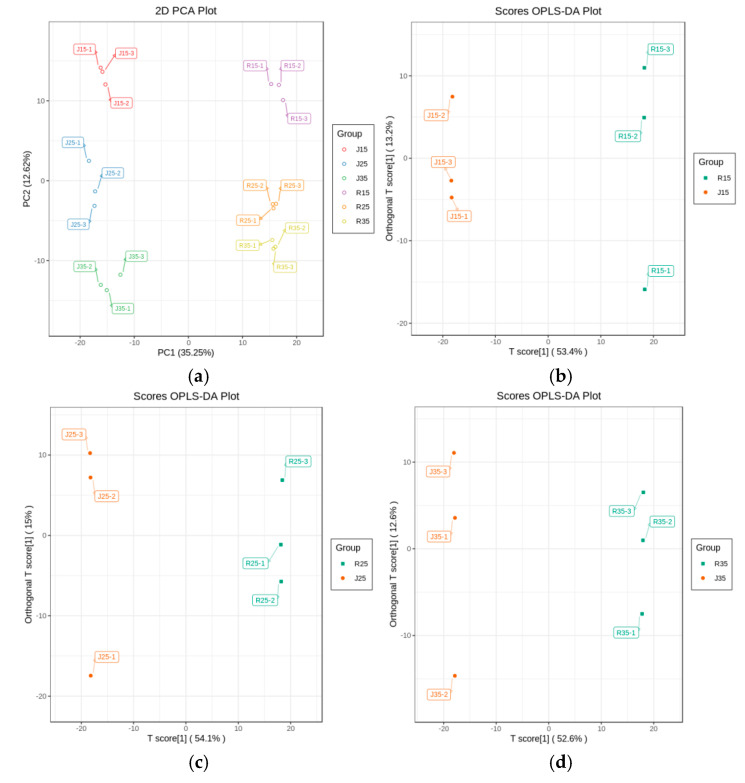
Multivariate statistical analysis: (**a**) PCA analysis; (**b**) OPLS−DA analysis at 15 °C; (**c**) OPLS−DA analysis at 25 °C; (**d**) OPLS−DA analysis at 35 °C.

The results of the pollen samples that were analyzed by PCA showed that the pollen samples under different temperature stresses had an obvious separation degree (Figure 4a). This variation reflected these metabolite changes of the two varieties (‘Renong No.1’ and ‘Jinhuang’) under different temperature stress. The results of OPLS-DA (Figure 4b–d) showed that these pollen samples of the two varieties (‘Renong No.1’ and ‘Jinhuang’) had significant spectral separation at each degree of temperature stress, indicating that the pollen metabolic differences of the two varieties under different temperature stresses were statistically significant.

### 3.3. Metabolites of Mango Pollen under Different Temperature Stress

**Figure 5:**
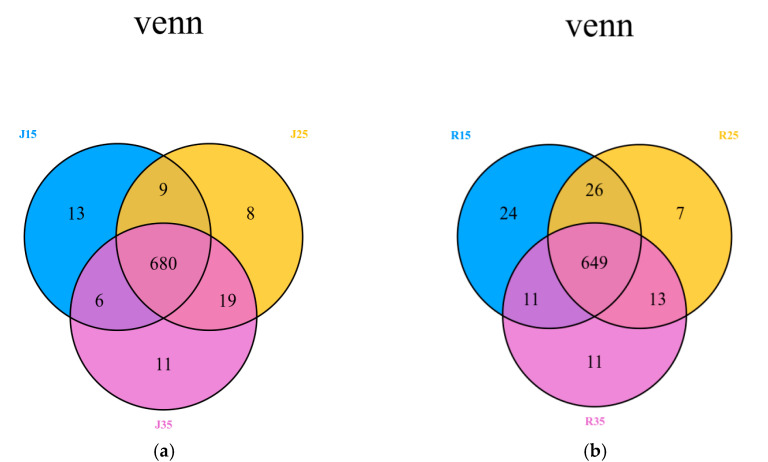
Venn diagram showing the overlapping and stage-specific differential metabolites under three temperature stresses (15 °C, 25 °C and 35 °C): (**a**) Jinhuang; (**b**) Renong No.1.

The chemical structure of the main chromatographic peak of the pollen samples was identified according to the peak area, retention time and accurate molecular weight of metabolites detected in LC-MS and the total particle flow chromatography. As shown in Figure 5, 18 samples were divided into six groups for metabolic analysis, and each group had three biological replicates. There were 775 metabolites detected based on the UPLC-MS/MS detection platform and self-built database. The number of different metabolites screened of the two mango varieties under three temperature stresses (15 °C, 25 °C and 35 °C) are shown in Figure 5. Among them, 680 metabolites (‘Renong No.1’: 649) were the same in all temperature gradient stages of ‘Jinhuang’. In addition, there were 9 metabolites (‘Renong No.1’: 26) under the 15 °C and 25 °C temperature stress, 6 metabolites (‘Renong No.1’: 11) under the 15 °C and 35 °C temperature stresses and 19 metabolites (‘Renong No.1’: 13) were found to be common under the 25 °C and 35 °C temperature stresses. However, 13, 8 and 11 metabolites (‘Renong No.1’: 24, 7 and 11) were specific only to the 15 °C, 25 °C and 35 °C temperature stress, respectively.

### 3.4. Metabolites Expression and Changes under Different Temperature Stresses

**Figure 6:**
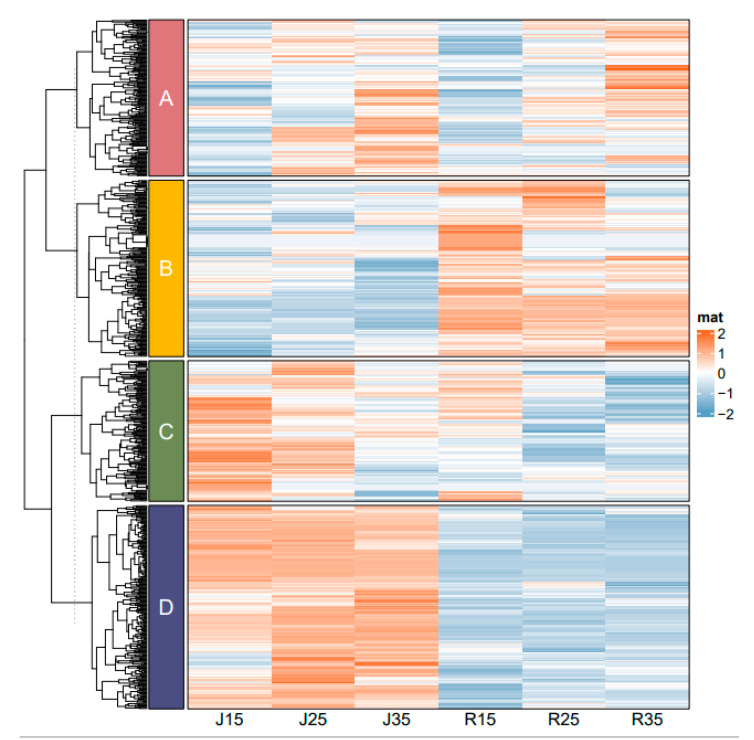
Heat map of the difference in pollen metabolite expression levels of two varieties (‘Renong No.1’ and ‘Jinhuang’) under different temperature stress. The thermogram shows the representative changes of heterometabolites in mango pollen germination at 15, 25 and 35 °C. A, B, C and D represents four categories of metabolites. The color marker strip on the right represents the value obtained after the standardization of the relative content of metabolites in the pollen samples (red represents high content and blue represents low content).

The expression of metabolites is shown in Figure 6. These results show that there are significant differences in the metabolites of mango pollen between the two varieties under different temperature stress. According to the expression of metabolites of the two mango varieties under different temperature stress, the metabolites were divided into four categories: A, B, C and D. These four categories of metabolites were divided into 12 categories of compounds (Table 2).

**Figure 7:**
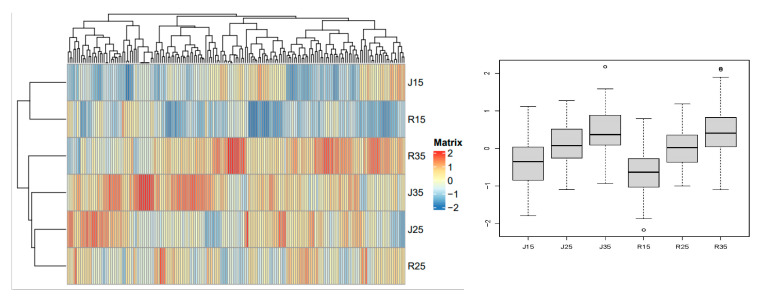
Class A metabolites of pollen in two varieties (Renong No.1 and Jinhuang). The heat map on the left shows the difference in the expression level of metabolites under different temperature stress. The heat map shows the representative changes of different metabolites in mango pollen germination at 15, 25 and 35 °C in vitro. The color marker strip on the right represents the value obtained after the standardization of the relative content of metabolites in the pollen samples (red represents high content and blue represents low content).

Class A metabolites contain 216 metabolites, which are mainly phenolic acids, flavonoids and esters. It can be seen from the heat map that the pollen metabolite contents of mango varieties ‘Renong No.1’ and ‘Jinhuang’ were significantly different under different temperature conditions of 15 °C, 25 °C and 35 °C. The box plots further showed the metabolite content distribution of ‘Renong No.1’ and ‘Jinhuang’ at different temperatures (15 °C, 25 °C, 35 °C). Both ‘Jinhuang’ and ‘Renong No.1’ had the highest metabolite content at 35 °C, followed by 25 °C, and the lowest metabolite content at 15 °C. For class A metabolites, temperature had a greater impact on metabolites, and the metabolite content was more similar at the same temperature between ‘Renong No.1’ and ‘Jinhuang’ (Figure 7). (Differential metabolites are shown in Supplementary Table S1).

**Figure 8:**
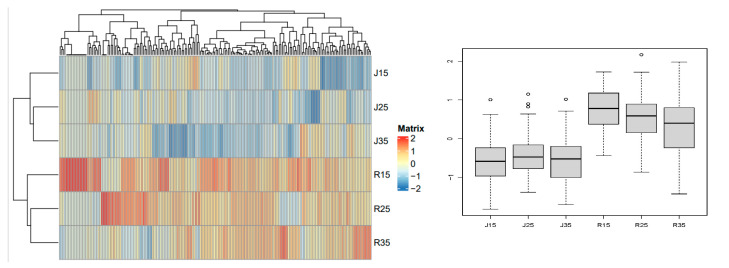
Class B metabolites of pollen in two varieties (Renong No.1 and Jinhuang). The thermogram on the left shows the difference in the expression level of metabolites under different temperature stress. The thermogram shows the representative changes of different metabolites in mango pollen germination at 15, 25 and 35 °C in vitro. The color marker strip on the right represents the value obtained after the standardization of the relative content of metabolites in the pollen samples (red represents high content and blue represents low content).

Class B metabolites contain 193 metabolites, which are mainly organic acids, lignans and coumarins, amino acids and their derivatives. The content of these metabolites in ‘Renong No.1’ was higher than in ‘Jinhuang’. The highest metabolite content of ‘Renong No.1’ pollen was at 15 °C, the next at 25 °C, and the lowest at 35 °C. However, the highest metabolite content of ‘Jinhuang’ pollen was at 25 °C, followed by 35 °C, and the lowest at 15 °C. For Class B metabolites, there were significant differences in the metabolite content between ‘Renong No.1’ and ‘Jinhuang’ (Figure 8). (Differential metabolites are shown in Supplementary Table S2).

**Figure 9:**
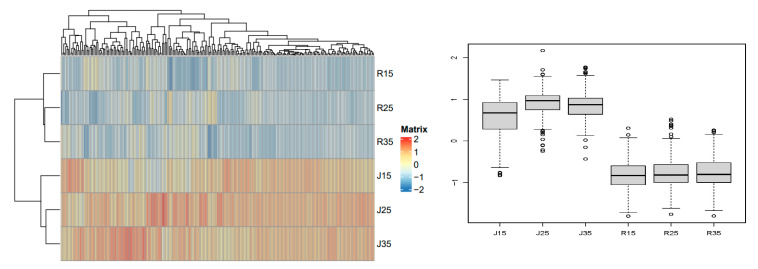
Class C metabolites of pollen in two varieties (Renong No.1 and Jinhuang). The thermogram on the left shows the difference in the expression level of metabolites under different temperature stress. The thermogram shows the representative changes of different metabolites in mango pollen germination at 15, 25 and 35 °C in vitro. The color marker strip on the right represents the value obtained after the standardization of the relative content of metabolites in the pollen samples (red represents high content and blue represents low content).

Class C metabolites contain 163 metabolites, which are mainly lipids, flavonoids and phenolic acids. The content of class C metabolites of pollen was significantly different between ‘Renong No.1’ and ‘Jinhuang’, and the content of metabolites in ‘Jinhuang’ was significantly higher than that in ‘Renong No.1’. For class C metabolites, under the three temperature stresses, metabolite contents did not differ significantly among the same varieties, and there were significant differences in metabolite contents between ‘Renong No.1’ and ‘Jinhuang’ (Figure 9). (Differential metabolites are shown in Supplementary Table S3).

**Figure 10:**
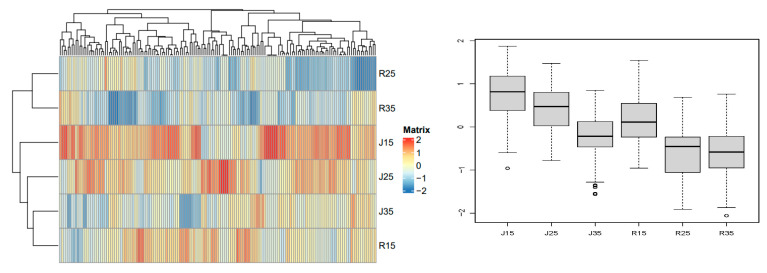
Class D metabolites of pollen in two varieties (Renong No.1 and Jinhuang). The thermogram on the left shows the difference in the expression level of metabolites under different temperature stress. The thermogram shows the representative changes of different metabolites in mango pollen germination at 15, 25 and 35 °C in vitro. The color marker strip on the right represents the value obtained after the standardization of the relative content of metabolites in the pollen samples (red represents high content and blue represents low content).

Class D metabolites contain 233 metabolites, which are mainly phenolic acids, flavonoids, and nucleotides and their derivatives. ‘Jinhuang’ had the highest metabolite content at 15 °C, followed by 25 °C, and the lowest metabolite content at 35 °C. Similarly, the highest metabolite content of ‘Renong No.1’ was at 15 °C, and the lower metabolite content was at 25 °C and 35 °C. For class D metabolites, there were significant differences in metabolite content between ‘Renong No.1’ and ‘Jinhuang’ (Figure 10). (Differential metabolites are shown in Supplementary Table S4).

### 3.5. Changes of Metabolites under Temperature Stress

**Figure 11:**
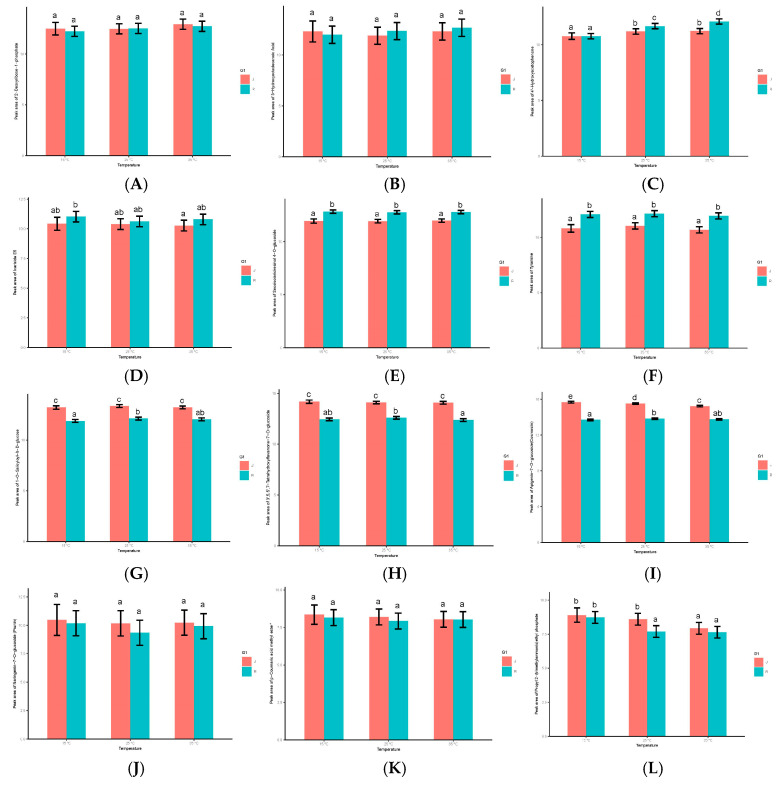
Histograms showing the differences in metabolites content in the pollen of ‘Jinhuang’ and ‘Renong No.1’ of Chinese fir at the three germination stages. (**A**) 2-Deoxyribose-1-phosphate, (**B**) Hydroxyoctadecanoic Acid and (**C**) Hydroxyacetophenone are category (**A**) metabolites; (**D**) Icariside E5, (**E**) Secoisolariciresinol 4-O-glucoside and (**F**) Tyramine are category (**B**) metabolites; (**G**) 1-O-Salicyloyl-β-D-glucose, (**H**) 3′,5,5′,7-Tetrahydroxyflavanone-7-O-glucoside and (**I**) Apigenin-7-O-glucoside (Cosmosiin) are category (**C**) metabolites; (**J**) Naringenin-7-O-glucoside (Prunin), (**K**) p-Coumaric acid methyl ester and (**L**) Propyl 2-(trimethylammonio) ethyl phosphate are category (**D**) metabolites. The lowercase letters on the bars represent the levels of significance. Means with the same lowercase letters at top of the bar did not differ significantly at *p* < 0.05.

A total of 12 different metabolites (three in each category) were randomly selected from four categories of metabolites. The content levels of selected metabolites under different temperature stresses are shown in Figure 11 (differential metabolites are shown in Supplementary Table S5).

From the metabolites of class (A), 2-Deoxyribose-1-phosphate, Hydroxyoctadecanoic Acid and Hydroxyacetophenone were selected as representatives; Icariside E5, Secoisolariciresinol 4-O-glucoside and Tyramine were selected from class (B) metabolites; 1-O-Salicyloyl-β-D-glucose, 3′,5,5′,7-Tetrahydroxyflavanone-7-O-glucoside and Apigenin-7-O-glucoside (Cosmosiin) were selected from class (C) metabolites; Naringenin-7-O-glucoside (Prunin), p-Coumaric acid methyl ester and Propyl 2-(trimethylammonio) ethyl phosphate were selected from class (D) metabolites. There were significant differences in the metabolite contents of these 12 metabolites in the pollen of the two varieties. In addition, the content changes of the 12 metabolites randomly selected were similar to the expression levels in the heat map.

### 3.6. Enrichment of Differential Metabolic Pathways

**Figure 12:**
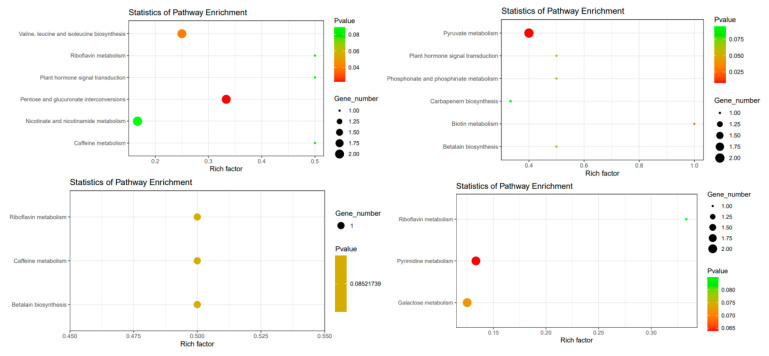
The enrichment analysis bubble chart shows the KEGG enrichment results of different metabolites. The top two figures show the comparison between 15 °C and 25 °C, and the bottom two figures show the comparison between 25 °C and 35 °C. The left is the comparison result of the ‘Renong No. 1’ variety, and the right is the comparison result of the ‘Jinhuang’ variety.

Different metabolites of the two mango varieties were mainly enriched in 13 metabolic pathways under different temperature stresses by liquid chromatography–mass spectrometry., including pyruvate metabolism, biotin metabolism, phosphonate and phosphinate metabolism, betalain biosynthesis, plant hormone signal transduction, carbapenem biosynthesis, riboflavin metabolism, galactose metabolism, pentose and glucuronate interconversions, valine, leucine and isoleucine biosynthesis, caffeine metabolism, nicotinate and nicotinamide metabolism and pyrimidine metabolism. These results are shown in Figure 12. During pollen germination under the different temperature stress, the metabolites of the two varieties of mango were differently enriched. Under the 15 °C temperature stress, the enrichment of ‘Renong No.1’ was the most obvious in pentose and glucuronate interconversions, followed by valine, leucine and isoleucine biosynthesis. ‘Jinhuang’ was most enriched in pyruvate metabolism. Under the 35 °C temperature stress, there was no significant difference in the enrichment metabolic pathway of ‘Renong No.1’ in betalain biosynthesis, caffeine metabolism and riboflavin metabolism. ‘Jinhuang’ was most enriched in pyruvate metabolism.

## 4. Discussion

Pollen is one of the manifestations of plant germplasm, which plays a pivotal role in plant cultivation, plant genetic breeding and germplasm resources conservation. Liu et al. [29] studied the germination and the length of the pollen tube of four mango varieties under different temperature stresses and their results suggested that the flowering period of mango was highly sensitive to changes in the external temperature; meanwhile, significant differences in pollen germination and pollen tube length were observed in different mango varieties. A low temperature or a high temperature stress can lead to changes in the pollen morphology, physiology and biochemistry, such as osmotic protectant accumulation, antioxidant synthesis, metabolic pathway etc. By comparing the metabolites of mango pollen of ‘Renong No.1’ and ‘Jinhuang’ treated under low temperature and high temperature stress, it was found that the metabolite contents of the two treatment groups at 15 °C and 35 °C changed significantly compared with that at 25 °C, and the pollen germination rate of ‘Renong No.1’ and ‘Jinhuang’ at the 25 °C temperature stress was significantly higher than that at the 15 °C and 35 °C temperature stress.

From the perspective of the overall metabolic pathway, the biosynthesis and enrichment of valine, leucine and isoleucine in ‘Renong No.1’ were the most obvious at the 15 °C temperature stress. ‘Jinhuang’ was most obviously enriched in pyruvate metabolism, indicating that the effect of low temperature on ‘Renong No.1’ was mainly concentrated in amino acid metabolism, and the effect on ‘Jinhuang’ was mainly related to glycolysis.

Amino acid was an important osmotic adjustment substance, which can directly or indirectly respond to abiotic press (temperature, water, saline-alkali etc.) in the environment [30,31] and its content can be commonly increased under low temperature stress [32]. Leucine, isoleucine and valine belong to branched chain amino acids due to their branched chain skeleton in the structure [33]. These kinds of amino acid can be generally accumulated under abiotic stress conditions including cold stress; therefore, they can be used as a protective penetrant [34,35]. The present study showed that amino acid content of ‘Renong No.1’ pollen was significantly increased under the 15 °C temperature stress, conjecturing that accumulating these amino acids was a strategy for ‘Renong No.1’ to maintain osmotic balance under low temperature stress. In addition, the biosynthetic enrichment of valine, leucine and isoleucine was the most obvious, indicating that ‘Renong No.1’ might have resistance to low temperature stress through osmotic balance adjustment. However, the main influence of high temperature stress at 35 °C on ‘Renong No.1’ focused on riboflavin metabolism, caffeine metabolism and betaine metabolism. Under high temperature stress, plant metabolism can change significantly. Multiple metabolites including lipids, sugars, organic acids, polyamines, amino acids etc. were all involved in plant response under stress [36,37,38]. When facing the stress of adverse environmental conditions, these metabolites played an important role in oxidation resistance, osmotic pressure adjustment, energy supply and cell membrane stabilization [34]. Riboflavin was widely involved in a series of reactions such as hydrogen, oxygen and electron transfer in plant basic metabolism; however, it can be synthesized in plants, which has a significant difference in different plants or organs. Riboflavin can combine with multiple polysaccharide to form glycosylated nuclide. Betaine plays various roles in adversity resistance such as oxygen free radical removal, permeability adjustment, biomembrance stabilization and photosynthesis adjustment to maintain the function and structure of enzymes and macromolecules. Moreover, betaine played an extremely important role in the resistance to high temperature stress [39]. Under the 35 °C temperature stress, ‘Renong No.1’ reassigned riboflavin and betaine to maintain a series of biological progress such as biomembrance stability and permeability, so as to regulate the environmental stress response.

Under the 15 °C temperature stress, the most obvious pathway of ‘Jinhuang’ enrichment was pyruvate metabolism. Glucose was degraded to pyruvate by enzyme catalysis, and accompanied by the formation of ATP. Therefore, ‘Jinhuang’ was regulated under low temperature stress through energy metabolism adjustment. Under the 35 °C stress, the most obvious pathway of ‘Jinhuang’ enrichment was pyrimidine metabolism, which is very important for plant metabolism and development [40]. Pyrimidine nucleotide plays a core role in the progress of information storage and extraction; it can act as DNA building blocks and a part of transcripts in nucleus or DNA synthesis organelle, playing an important role for division and elongation of tissue. Ribosome and transfer RNA are important parts of the protein synthesis mechanism, nucleotides are also used as direct precursors for the synthesis of B vitamins such as riboflavin, thiamine and folic acid [41], as well as some essential coenzymes such as nicotinamide adenine dinucleotide (NAD), flavin adenine dinucleotide (FAD) and sadenomethionine (SAM). In the basic energy progress of photosynthesis and respiration, purine nucleotide adenosine triphosphate (ATP) is produced with diphosphate phosphate and phosphate as the main triphosphate, which is used for general chemical energy saving. ATP is the main energy supplier for various metabolic reactions, and it can also be used as an activated precursor for synthesizing various macromolecules, including amino acids for protein synthesis and CIS glucose for starch synthesis. Thus, it can be inferred that the pollen of the ‘Jinhuang’ variety involved a complex body generation and extensive regulation process under the 35 °C temperature stress.

Different varieties had different metabolite changes under low and high temperature stress, which was related to its own biological characteristics. Thereinto, class A, C and D metabolites include flavonoids or flavonoid derivatives, indicating that flavonoid metabolites had an obvious regulation to mango pollen metabolism under different temperature stress. When facing different adversity stress, receptors in plant can be first activated, then downstream signal proteins such as receptor-like kinases (RLKs), mitogen-activated protein kinases (MAPKs) and heat shock factors (HSFs) can be activated, then genes related to the metabolic pathway can be activated, including chalcone synthases (CHSs), flavonol synthases (FLSs), UDP-dependent glycosyltransferases (UGTs), geranyl diphosphate synthases (GPSs), farnesyl diphosphate synthases (FPSs), geranylgeranyl pyrophosphate synthases (GGPSs) and terpene synthases (TPSs) involved in flavonoid metabolism, so as to promote the accumulation of flavonoids and terpenes and enhance the tolerance of plants to stress. Too much or too little reactive oxygen species are harmful and the content of reactive oxygen species depends on the delicate balance between production and antioxidant clearance [42]. Under abiotic stress, secondary metabolites play an important role to maintain the balance of ROS [43,44]. The more ROS produced, the higher flavonoid activity. The biological activity of these flavonoid compound can be regulated through their biosynthesis and mutual transformation, namely, by adjusting the content and proportion of flavonoids. The diversity of secondary metabolites plays an important role in the plant adapting to its environment. Flavonoid compounds have a buffering effect, which can accurately and quickly regulate the antioxidant capacity of cells and maintain the balance of reactive oxygen species through biosynthesis and transformation.

## 5. Conclusions

The pollen germination and pollen tube length of ‘Renong No.1’ and ‘Jinhuang’ reached the maximum at 24 °C–26 °C, which is of great significance for the cultivation and management of mango at the flowering stage. The metabolites at three different temperature stresses were clearly distinct between the two mango varieties ‘Renong No.1’ and ‘Jinhuang’. These results showed that 775 differential metabolites were screened by liquid chromatography–mass spectrometry and divided into 12 categories. There were significant differences in metabolite expression among different varieties and under different temperature stress, which was related to the biological characteristics of the varieties themselves. Among them, flavonoids or flavonoid derivatives were included in class A, C and D metabolites, indicating that flavonoid metabolites had an obvious regulatory effect on mango pollen metabolism under stress. The present study provided a new insight into how the metabolic differences of flavonoids under different temperature stresses affect pollen activity during the mango flowering period and further provided valuable information for reproductive biology studies and breeding in mango; in particular, the selection and breeding of the most suitable varieties for different production areas. Overall, we proposed potential strategies to breed heat- or cold-tolerant mango varieties and discussed the unique challenges that might be faced, which merits further investigation.

## Figures and Tables

**Table 1:** Information on the pollen samples and treatments.

Variety Name	Treatment Ⅰ	Treatment Ⅱ	Treatment Ⅲ	Treatment Time	Relative Humidity	No. of Replicates
Renong No.1	15 °C	25 °C	35 °C	3 h	60%	three
Jinhuang	15 °C	25 °C	35 °C	3 h	60%	three

**Table 2:** Classification of metabolites in pollen of the two varieties during pollen germination.

Category	Alkaloids	Amino Acids and Derivatives	Flavonoids	Lignans and Coumarins	Lipids	Nucleotides and Derivatives	Organic Acids	Others	Phenolic Acids	Quinones	Tannins	Terpenoids
A	14	18	25	4	23	6	24	32	33	10	13	14
B	14	24	8	25	12	20	27	34	1	1	13	14
D	8	25	50	7	12	28	25	21	50	1	5	1
C	2	6	29	3	46	8	7	21	28	8	3	2

## Data Availability

We have provided the raw metabolite data as Supplementary Information: supplementary raw metabolite data Table S1.

## Supplementary Materials

The following supporting information can be downloaded at: https://www.mdpi.com/article/10.3390/metabo14100543/s1. Figure S1. pollen germination of two varieties (‘Renong No.1’ and ‘Jinhuang’) under 15 °C, 25 °C and 35 °C temperatures stress respectively; Tables S1–S6: raw metabolite data.
