# Supplementary material for: Effects of Pollen Germination and Pollen Tube Growth under Different Temperature Stresses in Mango (Mangifera indica L.) by Metabolome
Source: Metabolites. 2024 Oct 11;14(10):543. doi: 10.3390/metabo14100543 (PMC11509298; doi:10.3390/metabo14100543)
Supplement: Supplementary file 1 [file metabolites-14-00543-s001.zip › Figure S1. pollen germination of two varieties (í«Renong No.1í» and í«Jinhuangí») under 15íμ, 25 íμ and35 íμ temperatures stress respectively.pptx]

## Slide 1
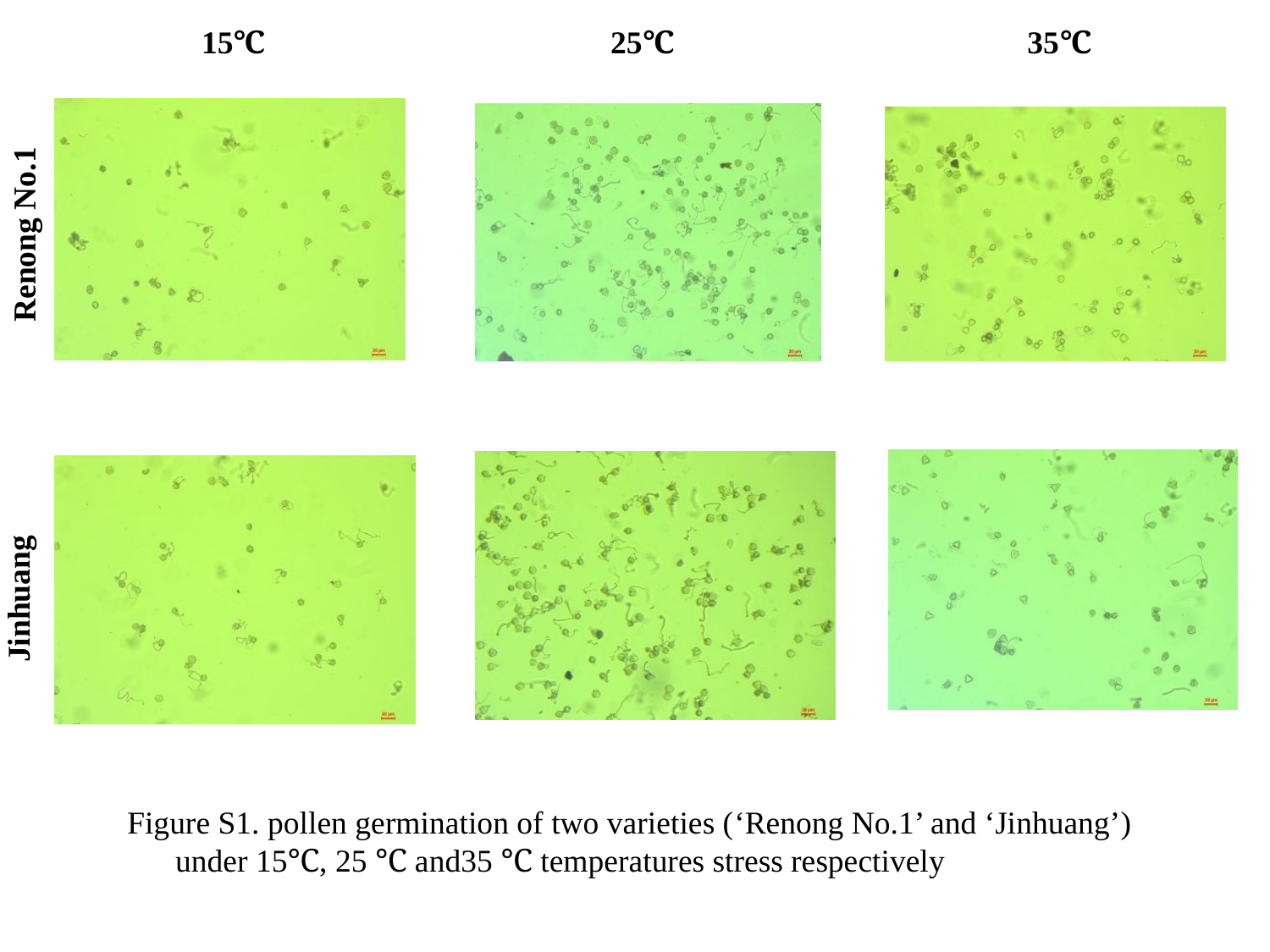

15℃ 25℃ 35℃
Renong No.1
Jinhuang
Figure S1. pollen germination of two varieties (‘Renong No.1’ and ‘Jinhuang’) under 15℃, 25 ℃ and35 ℃ temperatures stress respectively
